# Common variants in the *GNL3* contribute to the increasing risk of knee osteoarthritis in Han Chinese population

**DOI:** 10.1038/s41598-018-27971-4

**Published:** 2018-06-25

**Authors:** Bo Liu, Huiguang Cheng, Wenlong Ma, Futai Gong, Xiangyang Wang, Ning Duan, Xiaoqian Dang

**Affiliations:** 1 The First Department of Orthopaedics, the Second Affiliated Hospital of Xi’an Jiaotong University, Xi’an, Shaanxi China; 2 Department of Orthopedics and Traumatology, Xi’an Hospital of Traditional Chinese Medicine, Xi’an, Shaanxi China; 3 Department of Hip Joint, Honghui Hospital, Xi’an Jiaotong University, Xi’an, Shaanxi China; 4 Department of Hip Injury and Disease, Luoyang Orthopedic Hospital of Henan Province, Luoyang, Henan China; 5 Department of Traumatic Orthopaedics, Honghui Hospital, Xi’an Jiaotong University, Xi’an, Shaanxi China

## Abstract

Osteoarthritis (OA) is a complex degenerative joint disorder, which is caused by both environmental and genetic factors. Previous studies have indicated that the *GNL3* gene is associated with knee osteoarthritis (KOA) susceptibility in Europeans; however, the exact molecular mechanism is still unclear. In the present study, we investigated the potential genetic association of *GNL3* with KOA in a two-stage sample of 6,704 individuals from the Han Chinese population. Subjects containing 1,052 KOA patients and 2,117 controls were considered the discovery dataset, while subjects consisting of 1,173 KOA patients and 2,362 controls were utilized as the replication dataset. Single-SNP association, imputation, and haplotypic association analyses were performed. The SNP of rs11177 in *GNL3* was identified to be significantly associated with KOA after accounting for age, gender and BMI in both stages. The imputed SNP of rs6617 in SPCS1 was found to be strongly associated with KOA risk, and the significant association signal was confirmed in the replication stage. Moreover, a haplotype-based analysis also indicated a positive genetic effect of *GNL3* on KOA susceptibility. In summary, our results proved that *GNL3* plays an important role in the etiology of KOA, suggesting that *GNL3* is a potential genetic modifier for KOA development.

## Introduction

Osteoarthritis (OA) is the most common form of arthritis worldwide and is caused by degenerative changes in articular cartilage^1^. Severe OA influences victims’ quality of life and can even cause disability^2^. The World Health Organization (WHO) has estimated that more than 150 million people suffer from OA worldwide^3^. OA can occur in any joint, but knee OA (KOA) is the most common type. Among Chinese people aged over 60 years, the prevalence of symptomatic knee OA is 42.8% in women, which is higher than that in America^4^. Previous etiologic studies reported that environmental factors play an important role in the occurrence and development of KOA. Being overweight or obese, older and female can increase the risk factors and incidence of KOA^5–7^. However, a strong genetic component to osteoarthritis has been firmly established, with genetic factors estimated to account for up to 39–65% of the risk for developing KOA^8,9^. Hence, it is necessary to find the genes responsible for the susceptibility to KOA through candidate gene studies.

With the widespread application of sequencing and genetic association analysis for studying the genetics of complex diseases, candidate gene-based association studies have successfully mapped susceptibility for many complex diseases^10–16^. Many of the risk genes contributing to KOA susceptibility have been reported in previous studies. Among these candidate genes, the *GNL3* gene has gained much attention. The *GNL3* gene contains 15 exons spanning a 9 kb region located on 3p21.1, which encodes guanine nucleotide binding protein-like 3 that is important for stem cell proliferation, cell cycle regulation and maintenance of telomerase activity^17,18^. Recently, a genome-wide association scan with a subsequent replication study involving over 67,000 individuals (7,473 cases and 42,938 controls) of European ancestry produced highly significant evidence of the strong association of a single nucleotide polymorphism (SNP) rs11177 (C/T) with severe hip and knee OA (*P* value = 1.25 × 10^−10^), a missense polymorphism within the nucleostemin-encoding gene *GNL3*^19^. Moreover, allelic expression analysis has indicated that rs11177 in the *GNL3* gene is a cis-acting regulator in osteoarthritic cartilage, which contributes to the OA association signal at chromosome 3p21^20^. Furthermore, a previous study demonstrated that certain genes, including *SPCS1*, which surrounds *GNL3*, and *GNL3*, had an allelic expression imbalance in the same direction, with the minor allele producing fewer transcripts than the major allele^20^. These results further prove that *GNL3* may involve in the pathogenesis of OA.

To date, only two reports regarding the *GNL3* gene with OA are available, and both studies have shown that rs11177 in the *GNL3* gene is associated with OA in Europeans. Given that the underlying biological mechanism of *GNL3* contributing to KOA is still unknown and different ethnic populations may exhibit genetic heterogeneity of KOA, the replication of previous studies by using more samples from different populations would be needed. To the best of our knowledge, the association between *GNL3* and KOA has not been systematically evaluated in Han Chinese individuals. In the present study, we investigated the potential genetic association between *GNL3* and KOA to determine whether or not *GNL3* is associated with KOA in a Han Chinese population.

## Methods

### Subjects and measurements of clinical characteristics

Two independent datasets were included in this study, and a two-stage approach was utilized for the discovery of a single-marker analysis. Subjects including 1,052 KOA patients (aged 47–72 years) and 2,117 healthy age-matched controls (aged 47–72 years) were considered the discovery set and recruited from the Orthopedic Hospital of Henan Province (Luoyang city), while subjects including 1,173 KOA patients (aged 48–75 years) and 2,362 healthy age-matched controls (aged 48–75 years) were utilized as the replication set and enrolled from the Honghui Hospital of Xi’an Jiaotong University (Xi’an city). Two-stage subjects recruited in this study were random genetically unrelated Han Chinese individuals from the city of Luoyang in the Henan Province (the discovery dataset) and Xi’an in the Shaanxi Province (the replication dataset). The general characteristics of all two-stage subjects were collected, including age, gender and BMI. The characteristic information of the study subjects is summarized in Table 1. The diagnosis of KOA was confirmed by clinical examination and radiographic inspection. KOA was diagnosed by the symptomatic criteria of the American College of Rheumatology and the K-L grading standard. Subjects with a K-L grading of ≥2 at least in 1 knee were classified as OA cases. The healthy control subjects had no symptoms of arthritis or any other joint related disorders and with no family history of OA or other rheumatic diseases. Study subjects were excluded if they had arthropathy due to gout, pseudogout, rheumatoid arthritis and a history of other forms of arthritis, a history of knee surgery, secondary OA, knee trauma, other chronic inflammation diseases, and systemic or organic diseases. This study was performed in accordance with the ethical guidelines of the Declaration of Helsinki (version 2002) and was approved by the Medical Ethics Committee of Xi’an Jiaotong University. Informed consent was obtained from all subjects.

**Table 1:** The general characteristics of the subjects.

Characteristics	The discovery stage		*P*-value	The replication stage		*P*-value
	Cases (N = 1,052)	Controls (N = 2,117)		Cases (N = 1,173)	Controls (N = 2,362)	
Age (years, mean ± SD)	60.79 ± 7.32	61.12 ± 7.62	0.234	61.43 ± 8.04	60.88 ± 7.99	0.056
Gender (Female/Male)	601/451	1,217/900	0.848	640/533	1,297/1,065	0.831
BMI (kg/m^2^, mean ± SD)	26.26 ± 1.52	25.74 ± 1.51	**<*0***.***001***	26.42 ± 1.58	25.87 ± 1.55	**<*0***.***001***
K-L grading (%)						
Grade 2	506 (48.10%)	NA	NA	536 (45.69%)	NA	NA
Grade 3	319 (30.32%)	NA	NA	377 (32.14%)	NA	NA
Grade 4	227 (21.58%)	NA	NA	260 (22.17%)	NA	NA

BMI: body mass index; K-L: Kellgren-Lawrence; NA: not available.

### SNP selection and genotyping

SNPs in the *GNL3* gene were selected from the 1000 Genomes Chinese Han Beijing population (CHB) using Haploview. In total, 11 SNPs with minor allele frequencies (MAF) ≥ 0.01 were selected to be included in the study, including rs1108842, rs11177, rs3774349, rs117150867, rs35911561, rs183781382, rs75373137, rs35315313, rs13076193, rs6762813, and rs2289247. Genomic DNA was extracted from peripheral blood leukocytes according to the manufacturer’s protocol (Genomic DNA kit, Axygen Scientific Inc., California, USA). Genotyping was performed for all SNPs using the Sequenom Mass ARRAY RS1000 system (Sequenom, San Diego, California, USA). The results were processed using Typer Analyzer software (Sequenom), and genotype data were generated from the samples^21^. To ensure the accuracy of genotyping, we randomly chose 5% of our study subjects and repeated the genotyping process for those individuals. The concordance rate of this process was 100%, which indicated that the genotyping results of our study were reliable.

### Statistical Analysis

We examined the differences of some of the characteristic information between the cases and controls. χ^2^ test and Student’s t-test were performed for categorical variables and continuous variables, respectively. HWE was tested by Haploview v4.2 in both the discovery and replication stages. Single marker-based association analysis was conducted using Plink v1.9 in both stages. Logistic models were fitted for each SNP, and age, gender and BMI were included as covariates to eliminate the potential confounding effects. ORs and 95% confidence intervals (CI) were reported. Imputations based on genotype data were implemented through the IMPUTE2 software with 1000 Genomes Phase 3 CHB data as the reference panel. Subsequently, the association tests based on the imputed dosage data were conducted with the software SNPTEST v2. Notably, the parameter “average certainty” calculated in IMPUTE2 was employed as the main indicator of imputation quality. The threshold of indicator was chosen by exploring the patterns of Q-Q plots based on the *P*-values from association analyses of multiple marker sets obtained with different certainty thresholds. All significant SNPs in the discovery dataset and imputation analyses were included into the further analysis in the replication dataset. Linkage disequilibrium (LD) blocks were constructed using Haploview v4.2, and the haplotype frequencies were estimated by using GENECOUNTING v2.2. The difference of haplotypic frequencies between cases and controls were investigated, and haplotypic association analyses were conducted with a likelihood ratio test followed by permutation testing.

## Results

A total of 6,704 study subjects with Han Chinese ancestry were recruited, comprised of the discovery dataset (1,052 KOA patients and 2,117 controls) and the replication dataset (1,173 KOA patients and 2,362 controls). In Table 1, no significant differences for age and gender between the KOA patients and the controls were identified in both stages, but the KOA patients had a higher average body mass index (BMI) than the controls in both stages (*P* < 0.001). In patients of both stages, Kellgren-Lawrence (K-L) grading >2 accounted for more than 50% (Table 1). In the present study, a total of 11 SNPs within the *GNL3* gene were successfully genotyped in the discovery dataset. The results of the single SNP association analysis of 11 SNPs in the discovery stage, including the Hardy-Weinberg equilibrium (HWE) test, are summarized in Table 2 and Table S1. The HWE results indicated that the distribution of all SNPs genotypes were in HWE (*P* å 0.05) (Table 2 and Supplemental Table S1). As shown in Table 2, we identified association signals for the SNP rs11177 with KOA. The allelic *p* value was 0.000026 after accounting for the effects of age, gender and BMI. Further analysis also suggested that the A allele of rs11177 had a positive correlation with a risk of KOA (odds ratio (OR) = 1.25, adjusted by age, gender and BMI). Genotypic association analysis confirmed a similar pattern of results (Table 2).

**Table 2:** Associations of rs11177 and rs6617 with a KOA risk.

Group	H-WE *P*-value	Allele number (%)		Allelic *P*-value*	OR^*^ 95% CI	Genotype number (%)			Genotypic *P*-value*	
**The discovery stage**										
rs11177		G	***A***			GG	GA	AA	Co-dominant model	***0***.***000021***
Case	0.712	1056(50.19)	1048(49.81)	***0***.***000026***	1.25	268(25.48)	520(49.43)	264(25.1)	Dominant model	***0***.***001573***
Control	0.670	2362(55.79)	1872(44.21)		1.13–1.39	654(30.89)	1054(49.79)	409(19.32)	Recessive model	***0***.***000182***
**The replication stage**										
rs11177		G	***A***			GG	GA	AA	Co-dominant model	***0***.***000014***
	0.663	1181(50.34)	1165(49.66)	***0***.***000011***	1.25	301(25.66)	579(49.36)	293(24.98)	Dominant model	***0***.***000552***
	0.912	2640(55.88)	2084(44.12)		1.13–1.38	739(31.29)	1162(49.2)	461(19.52)	Recessive model	***0***.***000193***
rs6617		C	G			CC	CG	GG	Co-dominant model	***0***.***000834***
	0.335	1177(50.17)	1169(49.83)	***0***.***000222***	1.21	287(24.47)	603(51.41)	283(24.13)	Dominant model	***0***.***000552***
	0.934	2590(54.83)	2134(45.17)		1.09–1.33	709(30.02)	1172(49.62)	481(20.36)	Recessive model	***0***.***010504***

CI: confidence interval; OR: odds ratio.

Risk allele and significant *P* values are in bold italics. OR refers to the risk allele odds ratio in both groups.

*Means *P* values with adjustments for covariants (gender, age and BMI).

**Figure 1:**
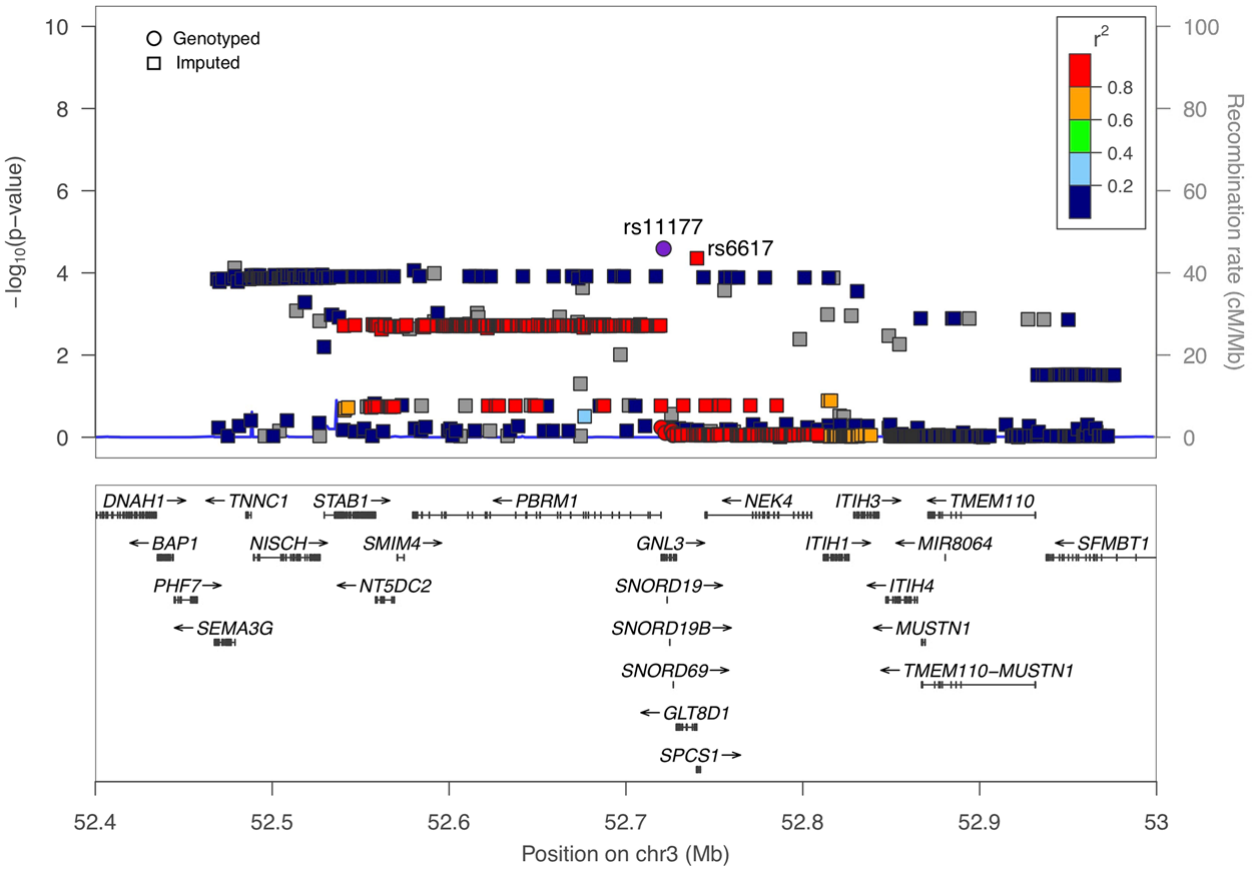
Regional association plots based on imputed region covering the region of *GNL3*. Imputed SNPs are indicated by circles, and genotyped SNPs are indicated by squares.

We conducted imputation for a 1 Mb genomic region, including *GNL3*. An average certainty threshold of 0.8 was determined to exclude imputed SNPs with low accuracy. This threshold was chosen by the pattern of Q-Q plots based on the *P*-values of the association analysis of multiple marker sets obtained at different certainty thresholds (Supplemental Figure S1). Another selection criterion was MAF ≥ 0.01 because our study focused on common variants. Based on the above filters, a total of 848 common variants were imputed with the reference panel and tested for association, and the results of the association analysis based on the imputations is also shown in Fig. 1. We summarize 391 associated SNPs imputed with the reference panel (*P* < 0.05) in Supplemental Table S2. However, only the significant association of rs6617 with KOA risk still remained after Bonferroni correction (*P* = 0.000044, *P*_*threshold*_ = 0.05/848 = 0.000059).

**Figure 2:**
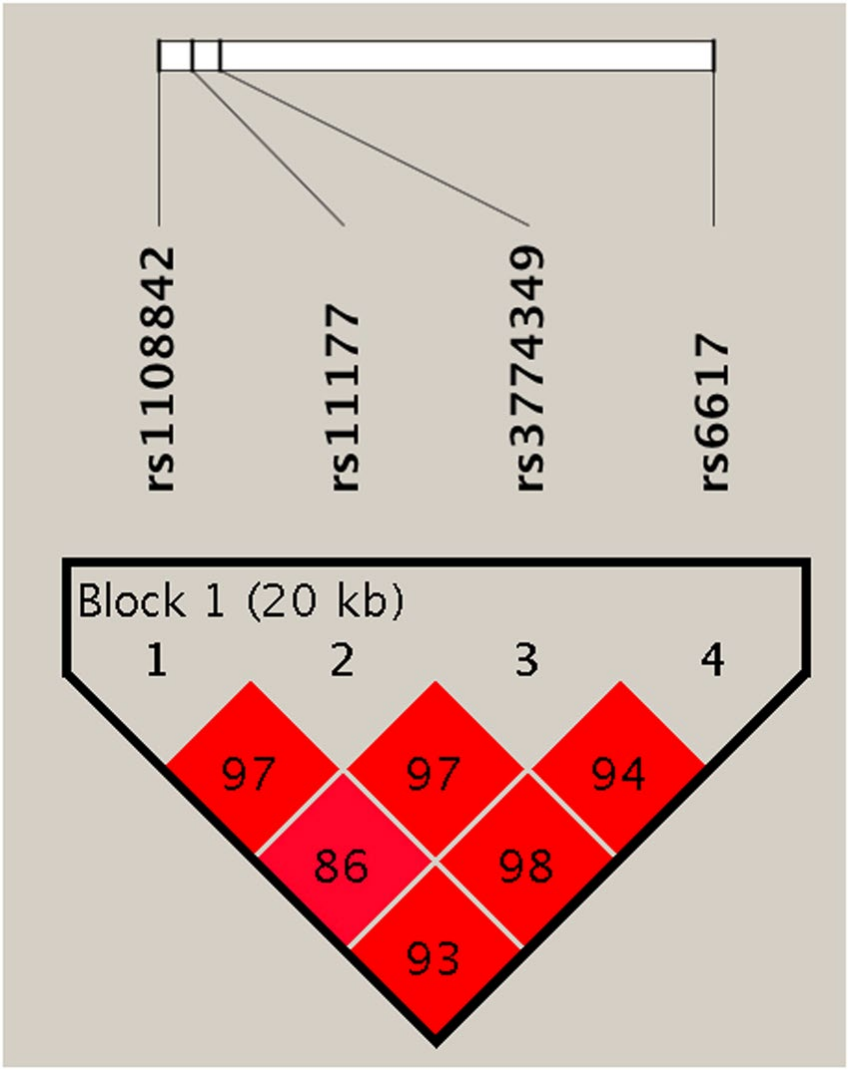
LD structure based on the replication stage data. The LD block is indicated by the shaded matrices.

Based on the imputation results and small effect sizes conferred by common alleles, which require the use of large samples, the overall state of a given SNP is best summarized by an association analysis from independent samples. Therefore, we performed single SNP association analysis for the SNP rs6617 and the SNP rs11177 together with another two SNPs (rs1108842 and rs3774349) within the same LD block (Supplemental Figure S2) in the replication dataset. The significant associations of rs11177 and rs6617 with a risk of KOA were replicated (Table 2). The other SNP still did not differ significantly in their allelic or genotype distributions (Supplemental Table S1). Based on the genotype data, we constructed the LD structure of genotyped SNPs in the replication stage. As shown in Fig. 2, one LD block is identified for four SNPs (rs1108842, rs11177, rs3774349 and rs6617). Haplotypic association analysis was performed to test the LD block, and a significant global *P* value (global *P* < 0.0001) was obtained from the LD block (Table 3). These results also indicated a positive genetic effect of *GNL3* on KOA susceptibility.

**Table 3:** Haplotype association analysis results based on the replication dataset.

Haplotype	Estimated frequencies (%)			Global *P-*value^**^
	Case	Control	*P*-value^*^	
**rs1108842-rs11177-rs3774349-rs6617**				
AGAC	48.54	46.35	0.0829	*** <0***.***0001***
***CACG***	46.98	43.68	***0***.***0172***	
***CGCC***	0.39	3.95	***<0***.***0001***	
AGCG	1.00	1.00	***0***.***9881***	

Haplotypes in bold italics are the significant ones in this study, and significant *P* values are in bold italics.

*Mean based on 100000 permutations.

**Mean based on comparison of frequency distribution of all haplotypes.

## Discussion

Previous studies have indicated that many common variants are involved in KOA susceptibility, but the exact mechanism of these genetic variants resulting in a risk of KOA remains unclear. Recently, the arcOGEN Consortium identified the SNP rs11177 in the *GNL3* gene to be significantly associated with OA in European populations through a genome-wide meta-analysis^19^. However, the role of the *GNL3* in the etiology of KOA is still unknown. The purpose of our study was to thoroughly examine the association of the *GNL3* gene with a risk of KOA in Han Chinese individuals. To our knowledge, this study is the first large-scale genetic association study of the *GNL3* gene with KOA susceptibility in Han Chinese individuals.

In our study, the SNP of rs11177 was identified to be significantly associated with KOA, with a risk A allele in the Han Chinese population in both stages. Next, we compared our results with those from the arcOGEN Consortium. In that study, a significant association was identified between SNP rs11177 and OA, including hip OA and knee OA. In both studies, the ORs were highly similar, and an A allele of rs11177 was the risk allele. However, both studies showed a significant difference in MAF at rs11177 between the Han Chinese population and Europeans, indicating that there is a certain degree of racial heterogeneity for the SNP rs11177. Moreover, the obvious differences may result from the examined common variants and the sample size of the different studies. Given that it is not sufficient to draw conclusions from limited SNPs analyses^22–24^, we performed a haplotype analysis. The haplotype analysis also showed a significant association of rs11177 with KOA in both stages, which provides further statistical evidence to further confirm the association of the SNP with a risk of KOA. Although our study had similar results to that reported by the arcOGEN Consortium, neither of the studies provided functional evidence of molecular mechanisms.

The *GNL3* gene encodes guanine nucleotide binding protein-like 3, also known as nucleoste (*NS*)^25^. This protein is expressed in mesenchymal stem cells, from which chondrocytes are derived^26^. NS plays an important functional role in cell cycle regulation and maintenance of telomerase activity^17,18^. It has been confirmed that the main pathological changes of KOA mostly focus on articular cartilage^1^. Previous studies indicated that the apoptosis of chondrocyte is closely related to telomere length and telomerase activity, and the excessive apoptosis of chondrocyte may lead to KOA^27^. Therefore, GNL3 may contribute to a risk for KOA through affecting telomerase activity in articular cartilage. In addition, several studies have demonstrated that NS mRNA transcription and protein expression levels are much higher in OA synovial and cartilage than in normal cartilage^28,29^. In animal experiments, NS levels were significantly higher in both the synovial tissue and cartilage of OA rat samples, and the expression of the *GLN3* gene was inhibited after treatment^30^. All of this evidence suggests that the *GLN3* gene is closely related to KOA, and it is reasonable to consider that *GNL3* might play an important role in the onset and development of KOA. Our results provide further supportive evidence for the association of the *GNL3* gene with KOA in the Han Chinese population. The SNP rs11177 with independent effects and some haplotypes in both stages were shown to be significantly associated with a diagnosis of KOA. Nevertheless, the molecular mechanisms by which *GNL3* affects KOA susceptibility remain unknown, and our results should be considered to be preliminary and cautiously interpreted.

Imputation has been considered as a powerful tool for fine-mapping untyped variants in candidate gene-based association studies^31^. Strict filtering criteria (certainty >0.8 and MAF ≥ 0.01) ensured the accuracy of imputed markers in our study. The information from the imputed SNPs provided us a novel approach to scrutinize our genetic data and identified an SNP of rs6617 in *SPCS1* associated with a risk of KOA. Given that there is an almost perfect LD between rs6617 and rs11177 (*r*^2^ = 0.98 in Asians and our subjects), it is reasonable to consider that the association signal from rs6617 in *SPCS1* should be the same with that from rs11177 in *GLN3*. *SPCS1* codes for signal peptidase complex subunit 1, much of which is still unknown. Gee *et al*. performed allelic expression analysis of six genes within and one gene close to the 3p21.1 and found that the expression of *SPCS1* was reduced in OA patients^20^. However, it is difficult to conclude that the decreasing expression of *SPCS1* could confer the risk of OA. On the other hand, *SPCS1* has been involved in the pathological changes of many diseases^32,33^, so further studies including more genes and more SNPs, especially functional ones, are necessary to clarify the potential effect of *SPCS1* on KOA risk.

There are several strengths in our study. Firstly, we have a large sample size (>6,000), which ensured sufficient power to capture the modest effects of SNPs. Secondly, we conducted a two-stage study design (discovery stage and replication stage from two different cities). With such a design, SNPs identified to be significant in both stages would be unlikely to be false positive. However, some limitations should also be noted. Hospital-based case-control studies always lead to selection sample bias. In addition, we only included genetic variations with MAF ≥ 0.01 for association analysis and cannot detect the potential contribution of rare variants because of lack of sufficient funds. Furthermore, we have tried our best to restrict population stratification when recruiting subjects by restricting the study subjects with a stable living environment^34,35^, but the potential population stratification could not be completely ruled out. These limitations might weaken the validity of our findings. However, similar conclusions drawn from two different ethnic groups (Han Chinese and European) demonstrated the genetic correlation between the *GNL3* gene and KOA.

In conclusion, we have shown that *GNL3* is an important locus for KOA and useful for an informative assessment of a genetic risk for KOA in Han Chinese individuals. Given the unknown complex network in the etiology of KOA, sequencing-based studies are needed in the future to investigate the genetic architecture of the genomic region of *GNL3* and its relationship with KOA-related phenotypes.

## Electronic supplementary material

Supplemental Materials
