# Supplementary material for: Common variants in the GNL3 contribute to the increasing risk of knee osteoarthritis in Han Chinese population
Source: Sci Rep. 2018 Jun 25;8:9610. doi: 10.1038/s41598-018-27971-4 (PMC6018215; doi:10.1038/s41598-018-27971-4)
Supplement: Supplementary file 1 — Supplemental Materials [file 41598_2018_27971_MOESM1_ESM.doc]

***Title:*** Common variants in the *GNL3* contribute to the increasing risk of knee osteoarthritis in Han Chinese population

***Author names and affiliations***: Bo Liu ^1,2^, Huiguang Cheng ^3^, Wenlong Ma ^4^, Futai Gong ^2^, Xiangyang Wang ^2^, Ning Duan ^5^ and Xiaoqian Dang ^1^

^1^ The First Department of Orthopaedics, the Second Affiliated Hospital of Xi’an Jiaotong University, Xi’an Shaanxi, China;

^2^ Department of Orthopedics and Traumatology, Xi’an Hospital of Traditional Chinese Medicine, Xi’an, Shaanxi, China;

^3^ Department of Hip Joint, Honghui Hospital，Xi’an Jiaotong University, Xi'an, Shaanxi, China;

^4^ Department of Hip Injury and Disease, Luoyang Orthopedic Hospital of Henan Province, Luoyang, Henan, China;

^5^ Department of Traumatic Orthopaedics, Honghui Hospital, Xi’an Jiaotong University, Xi'an, Shaanxi, China.

***Corresponding Author***:

Ning Duan, M.D. & Ph.D., Department of Traumatic Orthopaedics, Honghui Hospital, Xi’an Jiaotong University Health Science Center, No.555, Youyi East Road, Xi'an, Shaanxi, China, 710054.

Tel: 86-29-88418009; Fax: 86-29-62818386; E-mail: traunduan@163.com

Xiaoqian Dang, M.D. & Ph.D., The First Department of Orthopaedics, the Second Affiliated Hospital of Xi’an Jiaotong University, No.157, Xiwu Road, Xi'an, Shaanxi, China, 710004.

Tel: 86-29-87679292; Fax: 86-29-87679292; E-mail: [xjtuxqdang@163.com](mailto:xjtuxqdang@163.com)

Table S1 Allele and genotype frequency of single SNP association analysis

| SNP | | Allele Freq. (%) | | Allelic  *P*-value | Genotype Freq. (%) | | | Genotypic  *P*-value | H-W E  *P*-value |
| --- | --- | --- | --- | --- | --- | --- | --- | --- | --- |
| The discovery stage | | | | | | | | | |
|  | rs1108842 | A | C |  | AA | AC | CC |  |  |
| CASE |  | 1074(51.05) | 1030(48.95) | 0.586171 | 272(25.86) | 530(50.38) | 250(23.76) | 0.826444 | 0.794 |
| CTR |  | 2192(51.77) | 2042(48.23) |  | 569(26.88) | 1054(49.79) | 494(23.33) |  | 0.890 |
|  | rs3774349 | C | A |  | CC | CA | AA |  |  |
| CASE |  | 1074(51.05) | 1030(48.95) | 0.780543 | 271(25.76) | 532(50.57) | 249(23.67) | 0.961246 | 0.701 |
| CTR |  | 2177(51.42) | 2057(48.58) |  | 553(26.12) | 1071(50.59) | 493(23.29) |  | 0.561 |
|  | rs117150867 | G | A |  | GG | GA | AA |  |  |
| CASE |  | 1945(92.44) | 159(7.56) | 0.619488 | 899(85.46) | 147(13.97) | 6(0.57) | 0.883142 | 0.997 |
| CTR |  | 3899(92.09) | 335(7.91) |  | 1795(84.79) | 309(14.6) | 13(0.61) |  | 0.940 |
|  | rs35911561 | T | C |  | TT | TC | CC |  |  |
| CASE |  | 1958(93.06) | 146(6.94) | 0.703346 | 914(86.88) | 130(12.36) | 8(0.76) | 0.596540 | 0.161 |
| CTR |  | 3951(93.32) | 283(6.68) |  | 1844(87.1) | 263(12.42) | 10(0.47) |  | 0.850 |
|  | rs183781382 | C | T |  | CC | CT | TT |  |  |
| CASE |  | 1955(92.92) | 149(7.08) | 0.758091 | 908(86.31) | 139(13.21) | 5(0.48) | 0.848406 | 0.897 |
| CTR |  | 3943(93.13) | 291(6.87) |  | 1838(86.82) | 267(12.61) | 12(0.57) |  | 0.497 |
|  | rs75373137 | T | C |  | TT | TC | CC |  |  |
| CASE |  | 1951(92.73) | 153(7.27) | 0.635175 | 905(86.03) | 141(13.4) | 6(0.57) | 0.859113 | 0.842 |
| CTR |  | 3912(92.39) | 322(7.61) |  | 1810(85.5) | 292(13.79) | 15(0.71) |  | 0.394 |
|  | rs35315313 | G | T |  | GG | GT | TT |  |  |
| CASE |  | 1066(50.67) | 1038(49.33) | 0.673006 | 266(25.29) | 534(50.76) | 252(23.95) | 0.879461 | 0.618 |
| CTR |  | 2169(51.23) | 2065(48.77) |  | 553(26.12) | 1063(50.21) | 501(23.67) |  | 0.823 |
|  | rs13076193 | C | A |  | CC | CA | AA |  |  |
| CASE |  | 1058(50.29) | 1046(49.71) | 0.697794 | 263(25) | 532(50.57) | 257(24.43) | 0.911558 | 0.711 |
| CTR |  | 2151(50.8) | 2083(49.2) |  | 544(25.7) | 1063(50.21) | 510(24.09) |  | 0.836 |
|  | rs6762813 | C | T |  | CC | CT | TT |  |  |
| CASE |  | 1130(53.71) | 974(46.29) | 0.886506 | 306(29.09) | 518(49.24) | 228(21.67) | 0.984394 | 0.751 |
| CTR |  | 2282(53.9) | 1952(46.1) |  | 618(29.19) | 1046(49.41) | 453(21.4) |  | 0.791 |
|  | rs2289247 | G | A |  | GG | GA | AA |  |  |
| CASE |  | 1120(53.23) | 984(46.77) | 0.829116 | 298(28.33) | 524(49.81) | 230(21.86) | 0.959142 | 0.990 |
| CTR |  | 2266(53.52) | 1968(46.48) |  | 610(28.81) | 1046(49.41) | 461(21.78) |  | 0.751 |
| The replication stage | | | | | | | | | |
| CASE | rs1108842 | A | C |  | AA | AC | CC |  |  |
| CTR |  | 1201(51.19) | 1145(48.81) | 0.806371 | 306(26.09) | 589(50.21) | 278(23.7) | 0.964521 | 0.868 |
|  |  | 2433(51.5) | 2291(48.5) |  | 626(26.5) | 1181(50) | 555(23.5) |  | 0.965 |
| CASE | rs3774249 | C | A |  | CC | CA | AA |  |  |
| CTR |  | 1196(50.98) | 1150(49.02) | 0.630518 | 299(25.49) | 598(50.98) | 276(23.53) | 0.871934 | 0.493 |
|  |  | 2437(51.59) | 2287(48.41) |  | 621(26.29) | 1195(50.59) | 546(23.12) |  | 0.531 |

CTR: control

Table S2 The association test results of 391 imputed SNPs with significance

| SNP | Position | Allele A | Allele B | MAF | *P*-value |
| --- | --- | --- | --- | --- | --- |
| rs6617 | 52740182 | C | G | 0.46505 | 4.40E-05 |
| 3:52478783 | 52478783 | CGCACACAAACACTGCACAT | C | 0.064061 | 7.61E-05 |
| rs78691006 | 52580173 | A | G | 0.055517 | 8.69E-05 |
| rs146977835 | 52591637 | CT | CTT | 0.050752 | 0.000102 |
| rs6787493 | 52528000 | T | C | 0.048735 | 0.000112 |
| rs6445506 | 52528292 | T | C | 0.048739 | 0.000112 |
| rs887515 | 52523405 | C | T | 0.048694 | 0.000113 |
| rs6800707 | 52516293 | C | G | 0.048658 | 0.000113 |
| rs7620015 | 52510697 | G | A | 0.04866 | 0.000113 |
| rs728408 | 52517206 | G | A | 0.048661 | 0.000113 |
| rs4687543 | 52510982 | C | T | 0.048661 | 0.000113 |
| rs887514 | 52522659 | T | C | 0.048686 | 0.000113 |
| rs4687617 | 52509277 | G | A | 0.048634 | 0.000113 |
| rs11721286 | 52509029 | A | G | 0.048633 | 0.000113 |
| rs11928763 | 52508474 | A | G | 0.04863 | 0.000113 |
| rs11921116 | 52508406 | G | A | 0.048627 | 0.000113 |
| rs2109559 | 52512318 | A | G | 0.048676 | 0.000113 |
| rs6784615 | 52506426 | C | T | 0.048536 | 0.000114 |
| rs1011063 | 52506848 | C | T | 0.048553 | 0.000114 |
| rs2109558 | 52501616 | G | C | 0.048472 | 0.000115 |
| rs1541495 | 52492707 | T | C | 0.04838 | 0.000115 |
| rs2159607 | 52501451 | G | T | 0.048468 | 0.000115 |
| rs10865972 | 52491447 | C | A | 0.048358 | 0.000115 |
| rs34261027 | 52490391 | A | G | 0.048354 | 0.000115 |
| rs3066889 | 52488745 | TCA | T | 0.04834 | 0.000115 |
| rs1035002 | 52488202 | C | T | 0.048182 | 0.000115 |
| rs113966471 | 52478802 | T | C | 0.049613 | 0.000119 |
| rs138044951 | 52583325 | C | T | 0.045521 | 0.00012 |
| rs13099479 | 52677478 | G | A | 0.045586 | 0.00012 |
| rs35198201 | 52693366 | G | C | 0.045586 | 0.00012 |
| rs71084198 | 52670051 | GT | G | 0.045584 | 0.000121 |
| rs34735556 | 52611554 | G | T | 0.045532 | 0.000121 |
| rs111434579 | 52669498 | C | T | 0.045583 | 0.000121 |
| rs67976494 | 52614900 | CATT | C | 0.045533 | 0.000121 |
| rs13086936 | 52659079 | T | C | 0.045577 | 0.000121 |
| rs35225119 | 52623465 | A | T | 0.045532 | 0.000121 |
| rs71299614 | 52716943 | C | A | 0.04569 | 0.000121 |
| rs35272104 | 52618362 | G | C | 0.045531 | 0.000121 |
| rs71299610 | 52641703 | T | G | 0.045564 | 0.000121 |
| rs79993196 | 52697566 | T | C | 0.045595 | 0.000121 |
| rs35371456 | 52563462 | C | T | 0.045731 | 0.000121 |
| rs71299613 | 52698805 | G | A | 0.045595 | 0.000121 |
| rs145529151 | 52568641 | T | C | 0.045835 | 0.000121 |
| rs187869041 | 52568348 | C | T | 0.045809 | 0.000122 |
| rs7614424 | 52566354 | C | T | 0.045743 | 0.000122 |
| rs13066389 | 52565317 | C | T | 0.045743 | 0.000122 |
| rs13066644 | 52565247 | G | A | 0.045743 | 0.000122 |
| rs59021296 | 52560069 | GTGAC | GTGACTGAC | 0.045716 | 0.000122 |
| rs758802 | 52546116 | C | G | 0.045709 | 0.000122 |
| rs79979130 | 52551010 | C | T | 0.045713 | 0.000122 |
| rs35325270 | 52556369 | C | T | 0.045715 | 0.000122 |
| rs7644763 | 52546487 | C | T | 0.04571 | 0.000122 |
| rs10532068 | 52542845 | CAAT | C | 0.045667 | 0.000123 |
| rs758801 | 52536308 | A | C | 0.045209 | 0.000123 |
| rs4687545 | 52537969 | T | C | 0.045309 | 0.000124 |
| rs34182518 | 52743854 | G | A | 0.045197 | 0.000129 |
| rs13063138 | 52759254 | G | A | 0.045105 | 0.000129 |
| rs146545058 | 52758248 | AACACACAC | A | 0.045107 | 0.000129 |
| rs146729357 | 52756226 | T | C | 0.045111 | 0.000129 |
| rs13326165 | 52532118 | A | G | 0.044218 | 0.00013 |
| rs13087538 | 52763453 | G | A | 0.045089 | 0.00013 |
| rs45556336 | 52778572 | T | C | 0.045089 | 0.00013 |
| rs34131802 | 52800979 | G | A | 0.045054 | 0.00013 |
| rs67749743 | 52531681 | G | A | 0.044229 | 0.000131 |
| rs36012032 | 52814709 | C | A | 0.044879 | 0.000132 |
| rs9846089 | 52529773 | T | G | 0.044097 | 0.000132 |
| rs9863753 | 52529899 | A | T | 0.044138 | 0.000132 |
| rs372392661 | 52817149 | TGGGGGTCTCAGGC | T | 0.044838 | 0.000132 |
| rs199729098 | 52817164 | A | AC | 0.044837 | 0.000132 |
| rs6810027 | 52524574 | C | A | 0.043954 | 0.000133 |
| rs4687621 | 52524974 | A | C | 0.043954 | 0.000133 |
| rs4687544 | 52520502 | C | A | 0.043909 | 0.000133 |
| rs758803 | 52515533 | T | C | 0.043861 | 0.000133 |
| rs4687619 | 52518786 | T | C | 0.043882 | 0.000133 |
| rs9867823 | 52513027 | G | C | 0.043822 | 0.000133 |
| rs4687618 | 52513940 | T | C | 0.043836 | 0.000133 |
| rs11921913 | 52508534 | G | A | 0.04376 | 0.000134 |
| rs1011062 | 52506903 | A | G | 0.043672 | 0.000134 |
| rs1011064 | 52504503 | C | T | 0.043623 | 0.000135 |
| rs929529 | 52505525 | T | C | 0.043638 | 0.000135 |
| rs929528 | 52505445 | C | T | 0.043638 | 0.000135 |
| rs4687616 | 52501109 | T | C | 0.043577 | 0.000135 |
| rs6414568 | 52501292 | T | C | 0.043578 | 0.000135 |
| rs1035003 | 52504133 | G | T | 0.043618 | 0.000135 |
| rs6445483 | 52503701 | A | G | 0.043597 | 0.000135 |
| rs6445484 | 52503800 | A | G | 0.043597 | 0.000135 |
| rs6445486 | 52506491 | A | G | 0.043643 | 0.000135 |
| rs4312651 | 52506282 | A | G | 0.043642 | 0.000135 |
| rs6445485 | 52503823 | T | C | 0.043597 | 0.000135 |
| rs6771558 | 52497254 | C | G | 0.043531 | 0.000135 |
| rs6782162 | 52500452 | C | G | 0.043552 | 0.000135 |
| rs9847332 | 52500521 | T | C | 0.043552 | 0.000135 |
| rs6772191 | 52497849 | C | T | 0.043544 | 0.000135 |
| rs2335929 | 52498966 | A | G | 0.043546 | 0.000135 |
| rs9855470 | 52493275 | A | G | 0.043486 | 0.000135 |
| rs2335928 | 52498782 | A | C | 0.043546 | 0.000135 |
| rs3774409 | 52496229 | C | T | 0.043503 | 0.000135 |
| rs4687541 | 52495365 | G | T | 0.043502 | 0.000135 |
| rs13074649 | 52494141 | A | G | 0.043498 | 0.000135 |
| rs9850471 | 52492475 | A | G | 0.043461 | 0.000136 |
| rs4687614 | 52492085 | G | A | 0.043459 | 0.000136 |
| rs968588 | 52489564 | A | T | 0.043453 | 0.000136 |
| rs112686091 | 52489046 | T | TGG | 0.043452 | 0.000136 |
| rs2215466 | 52489074 | A | G | 0.043452 | 0.000136 |
| rs3066892 | 52489047 | A | G | 0.043452 | 0.000136 |
| rs3821838 | 52489049 | T | C | 0.043452 | 0.000136 |
| 3:52673015 | 52673015 | C | G | 0.070354 | 0.000136 |
| rs634382 | 52482851 | A | G | 0.044921 | 0.000139 |
| rs82825 | 52471942 | G | A | 0.044745 | 0.000139 |
| rs2016575 | 52477080 | T | C | 0.044787 | 0.00014 |
| rs613519 | 52468940 | T | C | 0.044713 | 0.00014 |
| rs3755810 | 52487100 | A | C | 0.04268 | 0.000142 |
| rs676034 | 52480446 | T | C | 0.039952 | 0.000163 |
| rs182689922 | 52470173 | T | C | 0.040147 | 0.000164 |
| 3:52675462 | 52675462 | C | CA | 0.0304 | 0.00023 |
| rs200229574 | 52755751 | CA | C | 0.09786 | 0.000265 |
| rs2240921 | 52830764 | C | T | 0.042314 | 0.00028 |
| 3:52518488 | 52518488 | A | C | 0.029159 | 0.000521 |
| 3:52518492 | 52518492 | A | C | 0.029159 | 0.000521 |
| 3:52513672 | 52513672 | CTCACCAGTCCCCATGCTGATAGCCA | C | 0.019645 | 0.000836 |
| 3:52593538 | 52593538 | G | C | 0.015208 | 0.000954 |
| 3:52615871 | 52615871 | C | A | 0.015208 | 0.000955 |
| rs187151675 | 52813971 | G | C | 0.015151 | 0.001032 |
| rs370690564 | 52533848 | G | C | 0.015044 | 0.001045 |
| 3:52533299 | 52533299 | G | T | 0.014997 | 0.001052 |
| rs138517812 | 52827401 | G | A | 0.014803 | 0.001101 |
| rs57215194 | 52662490 | A | ATTGT | 0.435273 | 0.001182 |
| rs187862454 | 52537776 | G | A | 0.024498 | 0.001209 |
| 3:52616427 | 52616427 | C | CT | 0.416116 | 0.00121 |
| rs148360193 | 52866622 | C | T | 0.018699 | 0.001282 |
| rs147786932 | 52884483 | C | T | 0.018699 | 0.001283 |
| rs143835121 | 52885570 | C | A | 0.018678 | 0.001284 |
| 3:52894069 | 52894069 | TC | T | 0.014272 | 0.001289 |
| 3:52927313 | 52927313 | T | C | 0.014055 | 0.001345 |
| 3:52936302 | 52936302 | A | G | 0.014011 | 0.001359 |
| rs148520461 | 52950111 | T | C | 0.018341 | 0.001379 |
| 3:52526842 | 52526842 | TTGTTGCTGTTGCTGTTGC | TTGTTGCTGTTGC | 0.088319 | 0.001478 |
| rs146977835 | 52591637 | CT | C | 0.450472 | 0.001532 |
| rs34959878 | 52672618 | C | CT | 0.446123 | 0.00158 |
| 3:52611446 | 52611446 | CAAAA | C | 0.46817 | 0.001749 |
| rs6414569 | 52708075 | A | T | 0.465002 | 0.0018 |
| rs4687624 | 52563572 | C | T | 0.463978 | 0.001803 |
| rs4434138 | 52556890 | A | G | 0.463962 | 0.001806 |
| rs4234633 | 52557038 | C | T | 0.463962 | 0.001806 |
| rs9758945 | 52604180 | A | G | 0.465041 | 0.001811 |
| rs71087003 | 52687605 | CA | C | 0.464934 | 0.001812 |
| rs35665793 | 52681932 | CT | C | 0.464934 | 0.001813 |
| rs11130313 | 52676190 | A | C | 0.464934 | 0.001813 |
| rs1961958 | 52585990 | A | G | 0.463322 | 0.001847 |
| rs10510760 | 52650348 | G | A | 0.463216 | 0.001852 |
| rs11295229 | 52596173 | TA | T | 0.460818 | 0.001869 |
| rs1133415 | 52575831 | G | A | 0.460821 | 0.001872 |
| rs2015971 | 52546820 | C | T | 0.459729 | 0.001872 |
| rs59021296 | 52560069 | GTGAC | G | 0.460941 | 0.001875 |
| rs13621 | 52558133 | T | C | 0.460939 | 0.001877 |
| rs5848957 | 52699346 | AT | A | 0.460633 | 0.001879 |
| rs67843199 | 52661018 | G | GA | 0.460678 | 0.001881 |
| rs4687625 | 52563718 | C | T | 0.459237 | 0.001909 |
| rs3774366 | 52641255 | T | C | 0.460119 | 0.001909 |
| rs11130308 | 52639257 | G | C | 0.460226 | 0.00191 |
| rs34005367 | 52558904 | A | T | 0.459237 | 0.00191 |
| rs4475032 | 52560021 | C | T | 0.459233 | 0.00191 |
| rs6790330 | 52632911 | G | A | 0.460228 | 0.00191 |
| rs4687637 | 52634092 | C | T | 0.460228 | 0.00191 |
| rs71615864 | 52633671 | CAA | C | 0.460228 | 0.00191 |
| rs9917834 | 52634619 | A | C | 0.460228 | 0.00191 |
| rs62253703 | 52632357 | T | C | 0.460228 | 0.00191 |
| rs10433615 | 52638482 | C | T | 0.460228 | 0.00191 |
| rs2083180 | 52668119 | G | T | 0.460128 | 0.001911 |
| rs6796726 | 52669389 | G | C | 0.460128 | 0.001911 |
| 3:52670773 | 52670773 | C | CAAAAA | 0.460128 | 0.001911 |
| rs13068293 | 52672167 | C | A | 0.460128 | 0.001911 |
| rs13098776 | 52670044 | C | T | 0.460128 | 0.001911 |
| rs11130310 | 52674667 | C | T | 0.460127 | 0.001911 |
| rs11130311 | 52675005 | T | C | 0.460127 | 0.001911 |
| rs11130312 | 52675055 | A | C | 0.460127 | 0.001911 |
| rs13086898 | 52675279 | G | A | 0.460127 | 0.001911 |
| rs13060675 | 52674300 | T | G | 0.460127 | 0.001911 |
| rs13081031 | 52674542 | C | T | 0.460127 | 0.001911 |
| rs4687633 | 52630548 | G | A | 0.460229 | 0.001911 |
| rs2164884 | 52629633 | C | T | 0.460229 | 0.001911 |
| rs4435633 | 52687562 | T | G | 0.460127 | 0.001911 |
| rs6770463 | 52686887 | G | A | 0.460127 | 0.001911 |
| rs2336149 | 52692124 | G | A | 0.460127 | 0.001911 |
| rs2590846 | 52692359 | C | G | 0.460127 | 0.001911 |
| rs11394906 | 52679288 | C | CT | 0.460127 | 0.001911 |
| rs13085775 | 52678370 | C | T | 0.460127 | 0.001911 |
| rs13085895 | 52678270 | G | C | 0.460127 | 0.001911 |
| rs13086297 | 52678494 | G | A | 0.460127 | 0.001911 |
| rs142853462 | 52680643 | CCAAGACAATT | C | 0.460126 | 0.001911 |
| rs7642198 | 52677960 | T | C | 0.460127 | 0.001911 |
| rs7611731 | 52692721 | T | C | 0.460127 | 0.001911 |
| rs2289250 | 52682946 | C | A | 0.460127 | 0.001911 |
| rs33967311 | 52683864 | C | A | 0.460127 | 0.001911 |
| rs34757451 | 52682205 | G | A | 0.460127 | 0.001911 |
| rs34754793 | 52629177 | A | T | 0.460232 | 0.001911 |
| rs7628578 | 52628321 | A | T | 0.460232 | 0.001911 |
| rs11717043 | 52656092 | A | T | 0.460118 | 0.001911 |
| rs34341238 | 52655415 | A | G | 0.460118 | 0.001911 |
| rs13069481 | 52666866 | C | T | 0.460115 | 0.001911 |
| rs4687548 | 52666650 | A | T | 0.460115 | 0.001911 |
| rs13064064 | 52657002 | T | C | 0.460118 | 0.001912 |
| rs150536093 | 52656367 | AAAAG | A | 0.460118 | 0.001912 |
| rs1561337 | 52659963 | G | A | 0.460118 | 0.001912 |
| rs34215106 | 52659124 | T | C | 0.460118 | 0.001912 |
| rs35249778 | 52661640 | G | C | 0.460118 | 0.001912 |
| rs4687639 | 52656340 | A | C | 0.460118 | 0.001912 |
| rs6445529 | 52662722 | T | A | 0.460118 | 0.001912 |
| rs7622694 | 52663882 | G | A | 0.460118 | 0.001912 |
| rs6804145 | 52694198 | C | T | 0.460126 | 0.001912 |
| rs12498066 | 52627660 | G | A | 0.460198 | 0.001912 |
| rs13085331 | 52627486 | G | C | 0.460198 | 0.001912 |
| rs2336146 | 52626646 | A | G | 0.460198 | 0.001912 |
| rs3852066 | 52621839 | C | T | 0.460198 | 0.001912 |
| rs1866268 | 52719398 | C | A | 0.460046 | 0.001912 |
| rs12488527 | 52698560 | T | C | 0.460075 | 0.001912 |
| rs62253700 | 52625019 | T | A | 0.460199 | 0.001913 |
| rs68021750 | 52624769 | G | A | 0.460199 | 0.001913 |
| rs10865974 | 52718280 | G | T | 0.460046 | 0.001913 |
| rs3733039 | 52719088 | C | T | 0.460046 | 0.001913 |
| rs7614498 | 52618941 | A | T | 0.460195 | 0.001913 |
| rs6445531 | 52717282 | T | C | 0.460052 | 0.001913 |
| rs34157897 | 52716937 | T | C | 0.460045 | 0.001913 |
| rs12637627 | 52619962 | G | T | 0.460201 | 0.001914 |
| rs12629699 | 52619792 | T | C | 0.460201 | 0.001914 |
| rs12629701 | 52619836 | T | C | 0.460201 | 0.001914 |
| rs11720243 | 52618018 | T | C | 0.460228 | 0.001914 |
| rs3755806 | 52643685 | T | C | 0.460124 | 0.001914 |
| rs12487591 | 52642936 | A | T | 0.460124 | 0.001914 |
| rs35189714 | 52642648 | CTT | C | 0.460124 | 0.001914 |
| rs3733045 | 52643307 | A | G | 0.460124 | 0.001914 |
| rs3774365 | 52642487 | T | C | 0.460125 | 0.001914 |
| rs11130315 | 52697163 | A | G | 0.460089 | 0.001915 |
| rs2336542 | 52710636 | A | G | 0.460038 | 0.001915 |
| rs11370862 | 52713306 | T | TC | 0.460069 | 0.001916 |
| rs62255362 | 52699741 | C | G | 0.46002 | 0.001917 |
| rs9714129 | 52711531 | T | C | 0.460028 | 0.001917 |
| rs71084190 | 52598668 | GA | G | 0.460234 | 0.001917 |
| rs33964154 | 52596914 | A | G | 0.460234 | 0.001918 |
| rs12637632 | 52708073 | A | T | 0.460093 | 0.001918 |
| rs13059862 | 52598540 | G | A | 0.460234 | 0.001918 |
| rs13060048 | 52598608 | G | A | 0.460234 | 0.001918 |
| rs1570 | 52586682 | T | A | 0.460233 | 0.001918 |
| rs17052256 | 52593119 | A | G | 0.460234 | 0.001918 |
| rs17052259 | 52593138 | A | G | 0.460234 | 0.001918 |
| rs3796353 | 52593230 | C | T | 0.460234 | 0.001918 |
| rs11419126 | 52593962 | G | GA | 0.460234 | 0.001918 |
| rs112363586 | 52710169 | T | A | 0.46004 | 0.001918 |
| rs62255368 | 52710172 | T | A | 0.46004 | 0.001918 |
| rs11130314 | 52697082 | T | C | 0.460092 | 0.001918 |
| rs11337750 | 52703254 | GA | G | 0.460045 | 0.001918 |
| rs2878628 | 52584715 | A | G | 0.460236 | 0.001918 |
| rs10433550 | 52603352 | A | G | 0.460231 | 0.001919 |
| rs34168767 | 52696798 | C | A | 0.460093 | 0.001919 |
| rs1821879 | 52600999 | T | C | 0.460231 | 0.001919 |
| rs13063160 | 52602274 | T | C | 0.460231 | 0.001919 |
| rs6788993 | 52605136 | T | C | 0.46023 | 0.001919 |
| rs6786919 | 52599789 | A | G | 0.460231 | 0.001919 |
| rs12496634 | 52709259 | A | C | 0.460095 | 0.001919 |
| rs67539070 | 52711974 | T | C | 0.460027 | 0.001919 |
| rs6786043 | 52604861 | A | G | 0.46023 | 0.001919 |
| rs6788887 | 52605005 | T | C | 0.46023 | 0.001919 |
| rs62255364 | 52701502 | C | T | 0.460025 | 0.00192 |
| rs77146033 | 52701501 | G | T | 0.460025 | 0.00192 |
| rs7652191 | 52610415 | T | C | 0.460221 | 0.00192 |
| 3:52609609 | 52609609 | AGCCTCTGCCTCT | AGCCTCT | 0.460223 | 0.00192 |
| 3:52609688 | 52609688 | AT | A | 0.460223 | 0.00192 |
| rs6805539 | 52609710 | T | A | 0.460223 | 0.00192 |
| rs4687547 | 52608949 | C | T | 0.460223 | 0.00192 |
| rs6762457 | 52609157 | G | A | 0.460223 | 0.00192 |
| rs6805156 | 52609362 | T | C | 0.460223 | 0.00192 |
| rs11714565 | 52606292 | C | T | 0.460222 | 0.00192 |
| rs4687546 | 52605943 | T | A | 0.460222 | 0.00192 |
| rs34140775 | 52606857 | C | CA | 0.460222 | 0.00192 |
| rs7625743 | 52606366 | C | G | 0.460222 | 0.00192 |
| rs5848958 | 52704076 | AG | A | 0.460039 | 0.001922 |
| rs4687630 | 52615301 | G | A | 0.460179 | 0.001922 |
| rs11720159 | 52617802 | T | G | 0.460189 | 0.001922 |
| rs11718509 | 52614670 | G | A | 0.460181 | 0.001923 |
| rs4687629 | 52615227 | G | C | 0.460181 | 0.001923 |
| rs34954168 | 52612159 | C | T | 0.460182 | 0.001924 |
| rs11718420 | 52611413 | T | C | 0.460182 | 0.001924 |
| rs1010552 | 52540544 | G | T | 0.455666 | 0.001932 |
| rs4687636 | 52633929 | G | A | 0.458496 | 0.001947 |
| rs34173654 | 52634605 | T | C | 0.458492 | 0.001947 |
| rs2336145 | 52629750 | A | C | 0.458497 | 0.001948 |
| rs2118540 | 52629386 | T | C | 0.458497 | 0.001948 |
| rs11708075 | 52624723 | C | T | 0.4585 | 0.001948 |
| rs4336110 | 52624233 | C | T | 0.458501 | 0.001949 |
| rs11714419 | 52628816 | A | G | 0.4585 | 0.001949 |
| rs10865973 | 52718154 | A | T | 0.458338 | 0.001951 |
| rs147587974 | 52718183 | CAAAAA | C | 0.458338 | 0.001951 |
| rs12488461 | 52698417 | T | C | 0.458389 | 0.001951 |
| rs12632265 | 52708288 | G | A | 0.458323 | 0.001952 |
| rs4130905 | 52709831 | A | T | 0.458373 | 0.001952 |
| rs35976524 | 52700477 | G | A | 0.458332 | 0.001954 |
| rs12487445 | 52618319 | A | C | 0.458506 | 0.001954 |
| rs13078422 | 52616978 | T | C | 0.458483 | 0.001954 |
| rs202187760 | 52717226 | T | TA | 0.458316 | 0.001955 |
| rs55678971 | 52717229 | T | A | 0.458316 | 0.001955 |
| rs13061253 | 52711472 | A | G | 0.458321 | 0.001955 |
| rs34610142 | 52708429 | G | A | 0.458375 | 0.001957 |
| rs12496476 | 52708737 | A | G | 0.458378 | 0.001958 |
| rs34537256 | 52657472 | A | G | 0.458381 | 0.001958 |
| rs62253733 | 52653414 | A | G | 0.458381 | 0.001958 |
| rs13065851 | 52644836 | T | A | 0.458381 | 0.001958 |
| rs11709448 | 52647449 | T | C | 0.458381 | 0.001958 |
| rs4687638 | 52651966 | A | C | 0.458381 | 0.001958 |
| rs71084197 | 52646903 | C | CA | 0.458381 | 0.001958 |
| rs35409678 | 52687686 | G | A | 0.458381 | 0.001959 |
| rs12486847 | 52691112 | T | C | 0.458381 | 0.001959 |
| rs13065019 | 52685244 | T | C | 0.458381 | 0.001959 |
| rs2336148 | 52690638 | G | A | 0.458381 | 0.001959 |
| rs3041438 | 52690944 | TAA | T | 0.458381 | 0.001959 |
| rs35211965 | 52685305 | C | G | 0.458381 | 0.001959 |
| rs62253740 | 52684128 | A | T | 0.458381 | 0.001959 |
| rs2028216 | 52680823 | C | T | 0.458381 | 0.001959 |
| rs201400694 | 52672993 | AT | A | 0.45838 | 0.001959 |
| rs11719514 | 52672531 | T | C | 0.45838 | 0.00196 |
| rs11720432 | 52672742 | T | C | 0.45838 | 0.00196 |
| rs6803012 | 52707026 | G | A | 0.458347 | 0.00196 |
| rs12493107 | 52706724 | G | T | 0.458347 | 0.00196 |
| rs6768697 | 52703615 | C | T | 0.458357 | 0.00196 |
| rs35107891 | 52613656 | G | C | 0.458464 | 0.001963 |
| rs17264436 | 52610651 | T | A | 0.458465 | 0.001963 |
| rs34115864 | 52612178 | A | G | 0.458464 | 0.001963 |
| rs11130307 | 52607682 | G | A | 0.458464 | 0.001963 |
| rs12632381 | 52607685 | G | A | 0.458464 | 0.001963 |
| rs7623199 | 52605974 | C | T | 0.458464 | 0.001964 |
| rs2289249 | 52597664 | G | A | 0.458465 | 0.001964 |
| rs143319527 | 52600064 | TCAAAA | T | 0.458464 | 0.001964 |
| rs6798246 | 52599922 | G | A | 0.458464 | 0.001964 |
| rs6778844 | 52596398 | T | C | 0.458468 | 0.001965 |
| rs6445528 | 52572447 | A | G | 0.455879 | 0.001983 |
| rs7638808 | 52572056 | A | G | 0.455874 | 0.001983 |
| rs2251219 | 52584787 | T | C | 0.455913 | 0.001985 |
| rs4687626 | 52569098 | G | A | 0.454258 | 0.002008 |
| rs12489732 | 52566820 | C | A | 0.454319 | 0.002022 |
| rs28661185 | 52567188 | G | T | 0.454225 | 0.002025 |
| rs13076398 | 52573096 | G | A | 0.454179 | 0.002025 |
| rs62256903 | 52573844 | G | A | 0.454179 | 0.002026 |
| rs150774614 | 52572841 | TTTG | T | 0.451706 | 0.002053 |
| rs1961959 | 52585760 | G | C | 0.449961 | 0.002087 |
| rs11719685 | 52676065 | C | T | 0.44723 | 0.002133 |
| rs3733046 | 52621627 | C | T | 0.443006 | 0.002201 |
| rs66995506 | 52577611 | CA | C | 0.43761 | 0.002303 |
| rs11711421 | 52561779 | C | T | 0.435136 | 0.002359 |
| rs199505925 | 52848590 | C | CT | 0.041883 | 0.003395 |
| 3:52798301 | 52798301 | CA | C | 0.039048 | 0.004108 |
| rs145664123 | 52854634 | AC | A | 0.050465 | 0.005467 |
| rs758800 | 52529266 | T | C | 0.137966 | 0.006348 |
| rs34605756 | 52696840 | TA | T | 0.480762 | 0.009775 |
| rs11705797 | 52959216 | G | A | 0.08124 | 0.030243 |
| rs4608686 | 52950554 | A | G | 0.081254 | 0.030263 |
| rs11713376 | 52958927 | A | G | 0.08124 | 0.030278 |
| rs11708337 | 52949254 | A | G | 0.081256 | 0.030284 |
| rs13095705 | 52958304 | C | T | 0.081241 | 0.030286 |
| rs3733035 | 52956023 | A | G | 0.08125 | 0.030289 |
| rs73839535 | 52954169 | T | C | 0.081252 | 0.030291 |
| rs11720488 | 52958590 | G | A | 0.081241 | 0.030293 |
| rs6762260 | 52953069 | T | C | 0.081252 | 0.030297 |
| rs3733034 | 52952433 | C | T | 0.081252 | 0.030298 |
| rs35569457 | 52949143 | T | G | 0.081255 | 0.030302 |
| rs13067373 | 52948461 | A | G | 0.081255 | 0.030303 |
| rs11716506 | 52944328 | C | T | 0.081246 | 0.030319 |
| rs11716612 | 52944645 | C | T | 0.081246 | 0.030321 |
| rs61473083 | 52957402 | T | C | 0.081247 | 0.03034 |
| rs56231111 | 52945786 | A | G | 0.081251 | 0.030352 |
| rs5848966 | 52948165 | G | GT | 0.081254 | 0.030363 |
| rs11718834 | 52963668 | A | C | 0.081212 | 0.030387 |
| rs11715231 | 52942776 | G | A | 0.081217 | 0.0304 |
| rs13068194 | 52942876 | C | T | 0.081217 | 0.030402 |
| rs10510761 | 52965255 | C | T | 0.081188 | 0.030412 |
| rs11715498 | 52943551 | G | C | 0.081217 | 0.030413 |
| rs13092352 | 52969697 | C | T | 0.081176 | 0.030423 |
| rs13062228 | 52932744 | T | C | 0.081244 | 0.030488 |
| rs35404467 | 52935763 | T | C | 0.08124 | 0.030495 |
| rs11717715 | 52975565 | G | A | 0.08116 | 0.030524 |
| rs11718538 | 52975987 | C | G | 0.08116 | 0.030525 |
| rs13069006 | 52973305 | G | C | 0.081162 | 0.030525 |
| rs11710707 | 52975916 | A | G | 0.08116 | 0.030529 |
| rs13074524 | 52974184 | A | G | 0.081161 | 0.030539 |
| rs2336541 | 52974630 | G | C | 0.081161 | 0.030547 |
| rs377291271 | 52974851 | GAAGTTAATTTTACCTGT | G | 0.081161 | 0.030552 |
| rs2336540 | 52974589 | G | A | 0.081161 | 0.030552 |
| rs184172684 | 52970504 | C | CT | 0.08117 | 0.030554 |
| rs11714037 | 52971680 | C | A | 0.081165 | 0.030582 |
| rs11713290 | 52971579 | G | A | 0.081168 | 0.030602 |
| rs34493168 | 52966821 | A | G | 0.081178 | 0.030627 |
| rs36126605 | 52937560 | G | A | 0.081234 | 0.030643 |
| rs57228349 | 52674201 | TCTGGTTATTATTCGC | T | 0.486325 | 0.049994 |

MAF: minor allele frequency


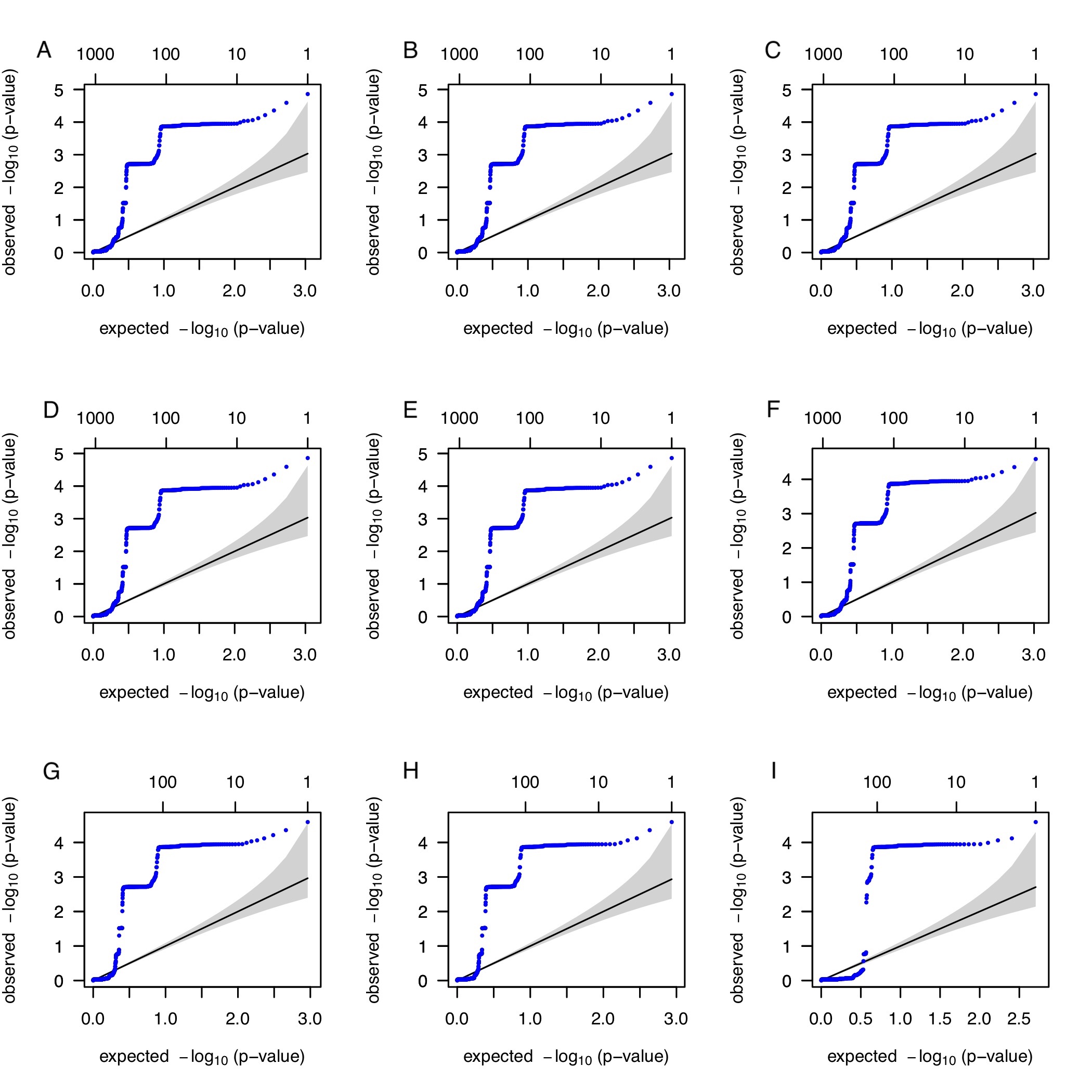


Figure S1. The Q-Q plots made by association test results based on marker set filter by different certainty thresholds with the reference panel of 1000 genomes CHB. The certainty thresholds used in a, b, c, d, e, f, g, h, i were 0.1, 0.2, 0.3, 0.4, 0.5, 0.6, 0.7, 0.8, 0.9 respectively. No significant deviance from expected line could be found when the certainty threshold was chosen as 0.8 in the imputations.


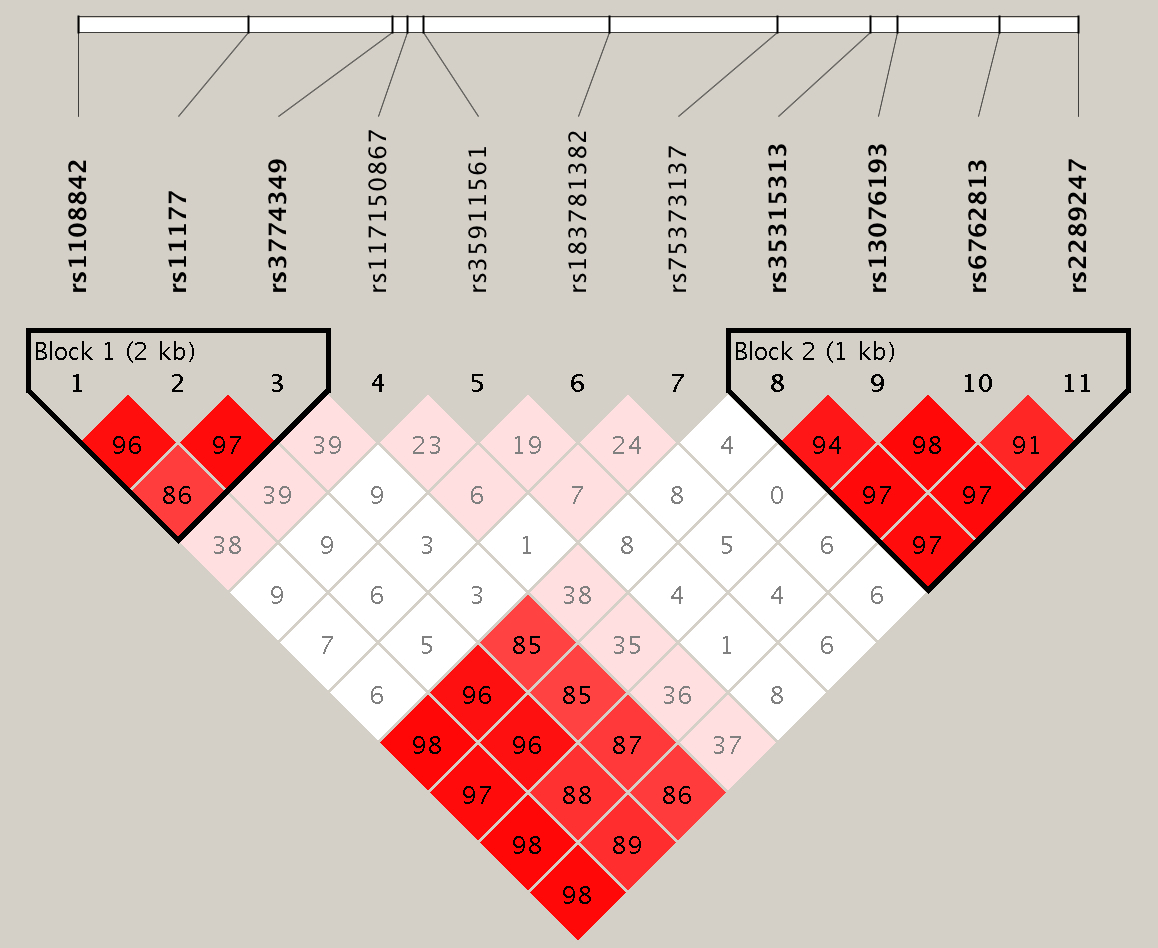


Figure S2. LD structure based on the discovery stage data**.** LD blocks were indicated by the shaded matrices.
